# Crashworthiness Design of Bidirectional Pyramidal Energy-Absorbing Tubes Based on Centipede Structures

**DOI:** 10.3390/biomimetics11010046

**Published:** 2026-01-07

**Authors:** Aodi Bie, Xiurong Guo, Danfeng Du, Yuchen Xie

**Affiliations:** 1 College of Home and Art Design, Northeast Forestry University, Harbin 150040, China; 2 College of Mechanical and Electrical Engineering, Northeast Forestry University, Harbin 150040, China; ddf72@nefu.edu.cn; 3 School of Mechanical Science and Engineering, Huazhong University of Science and Technology, Wuhan 430074, China; yuchenxie@hust.edu.cn

**Keywords:** foam-filled, impact load, centipede-inspired, bidirectional pyramidal tubes, energy absorption, crashworthiness

## Abstract

Energy-absorbing components should be effective and stable in engineering protective structure designs to reduce collision impacts. However, conventional energy-absorbing structures have considerable potential for optimization for energy dissipation and structural stability. Like other invertebrates, the centipede’s folding mode when moving forward is compatible with the hierarchical folding process when the energy-absorbing structure is impacted; however, this rule has not been thoroughly examined and proven. Based on this gap, this study built a unique biomimetic aluminum foam-filled bidirectional pyramid energy-absorbing structure, analyzed its geometric parameters on crashworthiness, and developed high-performance energy-absorbing components. Experiments and simulations were conducted on a bidirectional pyramid construction with three schemes for filling aluminum foam inspired by the centipede body section and profile. The construction with foam aluminum filling the gap has optimum specific energy absorption and load stability. Additionally, optimizing structural performance is most effective in certain ranges (78° ≤ *θ* ≤ 87°, *t* ≤ 0.1 mm, 34 mm ≤ *d* ≤ 44 mm). With Kriging and NSGA-III multi-objective optimization, the optimized peak crushing force decreases by 11.17% and specific energy absorption increases by 11.67%. The study and optimization process offers a theoretical reference for future high-performance energy-absorbing structures and has significant engineering application potential.

## 1. Introduction

In high-risk engineering fields such as vehicles, aviation and ships, the design of energy-absorbing structures is closely related to the safety of occupants because it often bears the last line of defense for accident energy dissipation [1,2,3,4,5]. However, existing thin-walled energy-absorbing structures commonly show critical bottlenecks under actual impact conditions: initial peak crushing force (IPCF) are difficult to effectively control, local buckling and deflection are common during folding, and energy absorption efficiency decreases rapidly during the mid-to-late stages of collision. As high-speed equipment requires more demanding safety standards, lightweight design, and resistance to impact reliability, the energy-absorbing capacity of conventional rectangular, polyhedral, and conical thin-walled types is reaching its limit. Their load-management capabilities and consistent energy absorption performance are unable to cope with the effect levels and structural restrictions imposed by new regulations [6].

To improve the energy absorption capacity of thin-walled structures, researchers usually fill the interior of hollow tubes with porous materials such as honeycomb structures [7,8], lattice structures [9,10], or metal foams [11,12]. Honeycomb structure has been widely concerned by researchers because of its regular arrangement of holes and excellent specific stiffness. It is often fabricated into metal sandwich panels such as aluminum alloy, stainless steel and titanium alloy and used in aviation, vehicle and protective engineering [13]. Rahimijonoush et al. [14] studied sandwich panels consisting of titanium faces and aluminum honeycomb cores at various projectile impact velocities. The results indicate that asymmetrical face thickness designs perform better against high-velocity impacts. Similarly, Sun et al. [15] performed a comprehensive analysis of the effect of geometric parameters such as aluminum plate thickness and honeycomb cell dimensions on collision responses. To improve the ability of structures to dissipate energy, honeycomb gaps were turned into lattice structures. These structures can be either two-dimensional (2D) or three-dimensional (3D) [16]. Two-dimensional (2D) stretched lattices and hierarchical 2D lattices are two common types of 2D structures. Three-dimensional structures are often made up of spatial arrays of cubic, pyramidal, rhombic dodecahedral, or octahedral cells. Wei et al. [17] used impact theory to construct and analyze the dynamic fragmentation behavior of star-triangular honeycomb (STH) auxiliary structures at various impact velocities. Hu [18] and He et al. [19] then proposed unit nesting and array combination methods that enhanced the energy absorption performance of 2D lattice matrices.

In contrast, foam metals, particularly foam aluminum, have received more attention in the recent past owing to their lightweight features and high specific energy absorption capacity, exceeding honeycomb and lattice structures [20]. The efficiency of foam aluminum is greatly impacted by the manufacturing process, notably the control of pore dimensions and pore structure parameters. Wang et al. [21] reviewed the influence of pore size of aluminum foam on the mechanical properties of the whole material in recent years, and found that the uniform distribution of pore size is more conducive to the coupling effect between foam and thin-walled tube. In view of this research direction, Zhang et al. [22,23,24] systematically studied the interaction mechanism between aluminum foam cores with different density gradients and thin-walled structures, and revealed the regulation effect of layered aluminum foam on the energy absorption behavior of structures. Furthermore, Googarchin et al. [25] proposed a prediction model of average compressive load when studying foam-filled conical tubes, which provided theoretical guidance for engineering design. In addition, Costas et al. [26] developed a composite collision box structure by combining aluminum tube, GFRP frame and PET foam core, which fully demonstrated the great potential of the synergistic effect of aluminum foam and composite materials in improving energy absorption performance. These studies have shown that metal foams not only have unique advantages in porous material systems but also provide core material support for the design of high-performance thin-walled energy-absorbing components.

Recently, biomimetic design has gradually become the mainstream research direction in the field of energy-absorbing structures. The natural mechanical structures in organisms such as microorganisms [27,28], plants [29,30], and animals [31] provide rich design inspiration for engineering protection systems and promote the development of innovative energy-absorbing structures. Based on the skeleton characteristics of deep-sea glass sponge, Xu et al. [32] proposed a biomimetic cylindrical sandwich structure (BCCS) with a counter-supporting lattice, which exhibits higher energy storage capacity under impact load. Luo et al. [33] drew inspiration from the natural spiral structure and constructed a foam-filled spiral tube (FFST), which can significantly reduce the load fluctuation of the structure in a high-load scenario, thereby improving its energy absorption stability. Similarly, Pham et al. [34] developed biomimetic multi-cell tubes by imitating the multi-cell configuration of the cross-section of bamboo cross-section, which showed excellent comprehensive impact resistance under axial and inclined loading. In contrast, Emre [35] considered the microporous voids within bamboo cross-sections and employed PLA material to fabricate tubular structures with varying grid patterns via 3D printing technology, subsequently conducting research on their energy-absorbing properties. Further, Yao et al. [36] proposed a bioinspired multicellular tube (BIMCT) based on the hierarchical structure of animal long bones. The lateral multi-cell layout and axial gradient foam filling inside the structure achieve the controllability of the crushing process. It is also found that the design of thickness gradient can improve the crushing stability of the structure, while the increase in average density will reduce the specific energy absorption (SEA) and cause the increase in IPCF. In addition, Wei et al. [37] proposed a biomimetic multi-cell tube configuration based on the microscopic fractal structure of beetle elytra, in which the B-type structure is the most prominent in the overall energy absorption performance. These studies show that the biomimetic design strategy can provide new design freedom in structural topology, material gradient and local strengthening mechanism, and provide a reliable engineering path for the development of more efficient and stable energy absorbers.

In the field of topological design and structural optimization, numerous studies recently have analyzed the force-bearing mechanisms of biological structures and simplified skeletal features, leading to the development of various innovative topological configurations with outstanding mechanical properties. Regarding mechanical performance enhancement, Yang et al. [38] analyzed the force transmission and tensile load-bearing capacity of mesh structures inspired by spider webs. Using topological optimization, they developed a biomimetic mesh fabric structure applied to engineering scenarios such as interception, capture, and transportation. In the field of energy-absorbing structures, Zhang et al. [39], inspired by the origami-like geometric features of tree branching regions, proposed a topological reconstruction of bark to create an OTSs structure for dissipating collision energy in railway vehicles. Beyond thin-walled structures, researchers modeled and optimized spatial topological features based on the multiscale fiber architecture of deep-sea glass sponges [40], proposing a biomimetic sponge metamaterial structure for architectural protection applications.

Although there have been a lot of studies in the field of porous materials and bionic structures, there are still some shortcomings in the existing work. The design of many bionic energy-absorbing structures only stays in the imitation of local morphology and lacks in-depth research on the multi-section coordination mechanism of organisms. In particular, the progressive folding mode and load transfer law of invertebrates such as centipedes in special environments have not been fully utilized. In addition, the research on the coupling effect between metal foam and bionic geometric shell is limited, and the influence of geometric parameters on energy absorption performance is not clear. Most of the current energy-absorbing structure designs rely on experience or local optimization and lack a systematic optimization framework based on high-precision surrogate models. In view of these shortcomings, this study proposes a bi-directional pyramid aluminum foam-filled composite energy-absorbing structure based on the centipede folding mode. Combined with experiments and finite element analysis, its deformation and energy absorption mechanism are revealed, and Kriging (KRG)-NSGA-III multi-objective optimization research is carried out. This study aims to fill the gap in the coupling optimization design of bionic folding mechanism and porous materials and provide new strategies and application basis for anti-collision energy-absorbing structure and lightweight design.

## 2. Design of Bionic Energy Absorbers

### 2.1. Similarity Analysis Between Centipedes and Energy Absorbers

**Figure 1:**
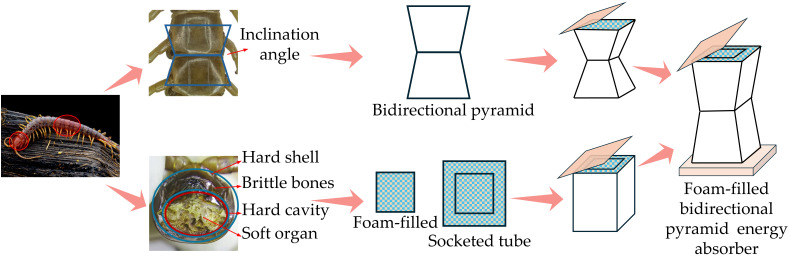
Structural feature extraction of centipede truck and head.

The centipede is a typical segmented-structure invertebrate, and its body shape shows unique mechanical characteristics. Figure 1 illustrates how multiple bidirectional conical segments connect the centipede’s torso. According to relevant research, the centipede rigidly draws the flexible body under rugged terrain conditions for adaptive bending and contraction, thus forming a folding dynamics model of the muscle group in the body [41,42]. In addition, the multi-layer structure of the centipede head, including a hard shell, brittle bone, cavity tissue, and soft organ, effectively enhances the cushioning ability of the centipede to external shocks through the design of a “hard-soft combination.” This impact resistance mechanism is not just a morphological analogy but the functional characteristics of the centipede segment structure in response to external forces, which can effectively prevent damage to key parts [43]. Therefore, the structural design of the centipede has a clear anti-impact feature in function, and this feature is optimized through natural selection, not just a conceptual imitation of biological morphology.

Similar to the ‘hard-soft combination’ principle of the centipede, the energy-absorbing structure effectively absorbs the impact energy through its own unique structural design during the collision process, thereby avoiding damage to the main bearing part. Energy-absorbing boxes dissipate energy using deformation methods such as bending, collapse, and fracture. This procedure is equivalent to the method by which centipedes distribute tension when exposed to outside influences by folding and deforming their segments.

In relation to force direction, the energy-absorbing box absorbs the majority of contact loads from the front, whereas the centipede also defends against front-facing dangers during movement to protect crucial parts from harm. From a macro perspective, the centipede’s trunk consists of multiple bidirectionally tapered segments connected together, exhibiting internal structural characteristics similar to porous materials. Impact-absorbing energy-dissipation boxes often incorporate lightweight porous materials within thin-walled structures to enhance energy absorption. As a result, the segmented composite structure of centipedes has energy-absorption mechanisms that are similar to those of energy-dissipation boxes. This biomimetic structure offers innovative insights into energy-dissipation design, meeting mechanical functional requirements while providing effective energy-absorption pathways (as shown in Figure 1).

### 2.2. Energy-Absorption Model Based on Centipedes

**Figure 2:**
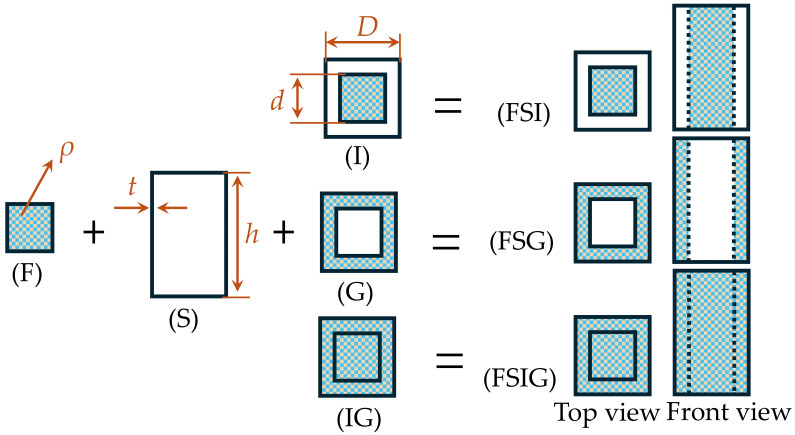
Bionic centipede-inspired square tube structure components.F stands for foam aluminum, S for square tubes, I for interior filling, G for gap filling, and IG for interior and gap filling.

**Figure 3:**
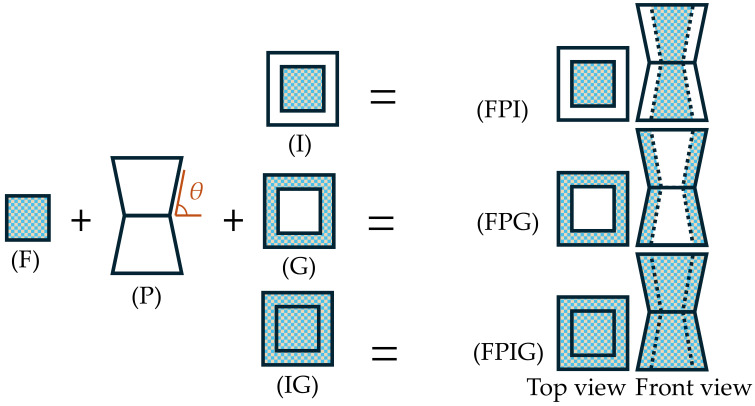
Bionic centipede bidirectional pyramidal structure components. F stands for foam aluminum, P for bidirectional pyramid tubes, I for interior filling, G for gap filling, and IG for interior and gap filling.

The centipede body is made up of several protein-based porous structures. In challenging conditions, these structures combine with the chitinous exoskeleton on the body’s surface, thus boosting the structural impact resistance. Inspired by the above, this work uses porous materials in a biomimetic impact-resistant box design to replicate the body-core structure (Figure 2 and Figure 3 (F)). At the same time, researchers in the field of porous materials have shown a lot of interest in foam aluminum as a new material for energy absorption in recent times [44].

When a centipede faces an obstruction ahead, its body segments deform preferentially at their joints, generating the principal deformation and folding zones. As shown in Figure 1, when the centipede folds, a folding segment progressively arises from the center to both sides. Inspired by this, this research proposes a biomimetic impact-resistant box with a shell that has a bidirectional pyramidal structure with a center depression. In Figure 3 (P), the side indentation angle is set at *θ*. For instance, Figure 2 (S) shows a tubular arrangement without a center indentation.

Furthermore, an analysis of the centipede’s head cross-section suggests that its epidermis, protein cells, and cartilage tissue display a functional synergy of “rigid load-bearing–flexible energy dissipation–rigid load-bearing–flexible energy dissipation,” hence increasing its overall impact resistance. To verify its structural properties, this study used a nested bidirectional pyramidal tube construction within another bidirectional prismatic tube. To explore the effect of various filling ways on energy absorption performance, foam aluminum was filled in three different layouts: just the center, only the gaps, and both the center and the gaps (as illustrated in Figure 2 and Figure 3, (I), (G), and (IG)).

In alignment with the aforementioned design concept, six experimental specimens were constructed (Figure 2 and Figure 3), namely: foam-filled square tube interior (FSI), foam-filled square tube gap (FSG), foam-filled square tube interior and gap (FSIG), foam-filled bidirectional pyramidal interior (FPI), foam-filled bidirectional pyramidal gap (FPG), and foam-filled bidirectional pyramidal interior and gap (FPIG). The outer tube diameter (*D*) and height (*h*) of all components were uniformly set at 80 mm and 120 mm, respectively, according to the parameters used by Li et al. in their study on energy-absorbing structures [45]. The inner tube diameter (*d*), wall thickness (*t*), inclination angle (*θ*), and foam aluminum density (*ρ*) were set at 48 mm, 1 mm, 85°, and 0.3 g/cm^3^, respectively. The range of *d* values was obtained by proportionately scaling the centipede’s head cross-sectional sizes (7.12 mm and 4.23 mm). The tilt angle *θ* was rounded to 85° based on the measured folding angle of 83.51° for the centipede’s torso. Given the mechanical properties and energy absorption characteristics of foam aluminum at different densities, a common value of 0.3 g/cm^3^ was selected for superior performance [46,47].

### 2.3. Evaluation Indicators

Energy-absorbing components are often put in front of the mechanical frame for the purpose to sustain stresses during operation and collisions. When outside hits surpass a certain limit, the structure absorbs energy via deformations like buckling and collapse, reducing primary structural damage. To precisely assess the impact resistance of the biomimetic bidirectional pyramidal energy-absorbing structure, accurate crashworthiness evaluation criteria must be devised. Common performance criteria include energy absorption (EA), SEA, IPCF, mean crushing force (MCF), and crushing force efficiency (CFE). These factors correspond to a structure’s energy absorption capacity, energy usage per unit mass, impact resistance, and energy absorption stability. Their calculating techniques are described in the Supplementary Materials (Equations (S1)–(S4)).

## 3. Crushing Response of Bionic Centipede Structures

### 3.1. Simulation Setup

**Figure 4:**
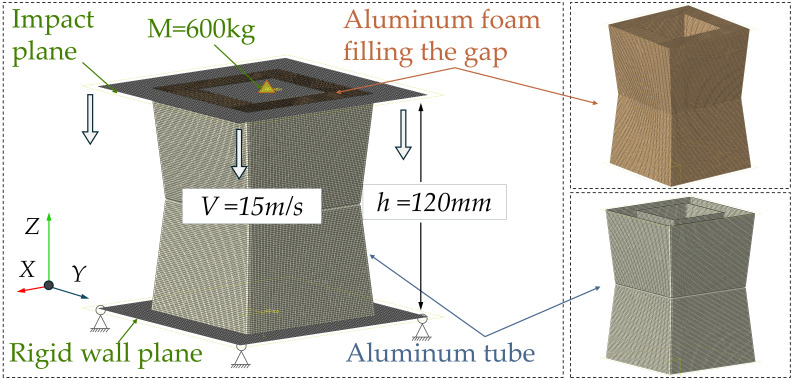
Simulation model.

To further investigate axial impact, a simulation model was established using the finite element tool Abaqus/Explicit, as shown in Figure 4. The bottom surface of the thin tube is connected to the lower rigid plate, with degrees of freedom constrained in all directions; the top surface contacts the upper rigid plate. In the simulation, based on previous studies, an initial axial impact load of 15 m/s was applied along the component direction, and a point mass of 600 kg was added to the upper rigid plate [46,48]. Simultaneously, all degrees of freedom except the axial direction of the thin tube were constrained. The friction coefficient is set to “hard contact” in the normal direction, while a penalty function of 0.25 is applied in the tangential direction. Furthermore, the model employs a general contact type to account for structural interactions.

**Figure 5:**
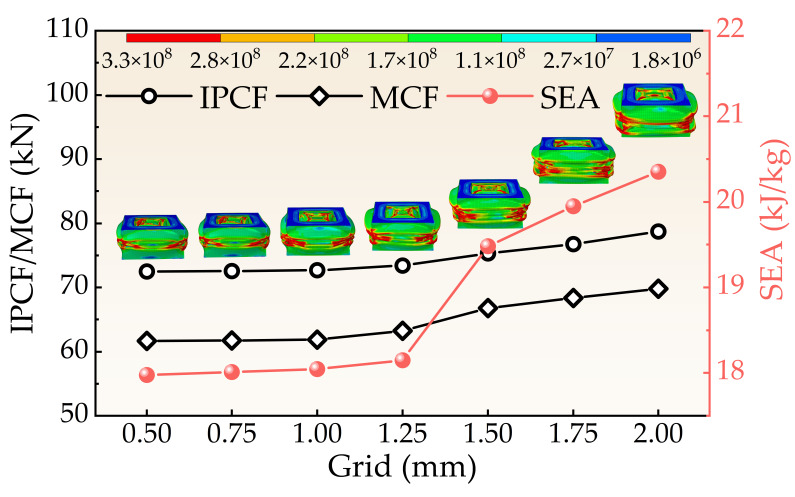
Mesh sensitivity analysis of the FPG structure.

Thin tubes were meshed using the S4R shell element (4-node double-curved thin or thick shell, reduced integration, hourglass control, finite membrane strain). Considering the potential for large deformations during impact, a mesh size sensitivity analysis was conducted, as shown in Figure 5. A 1 mm element size was ultimately selected, ensuring simulation accuracy while enhancing computational efficiency. Convergence performance with this element size was comparable to that of a 0.5 mm mesh, leading to its adoption for the entire structural modeling.

**Figure A1:**
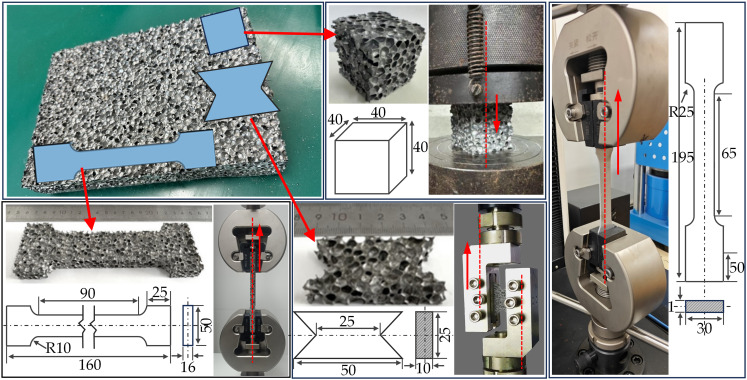
Experimental device and sample schematic diagram of material performance test (foam compression, tensile, shear device, aluminum alloy tensile device).

**Figure A2:**
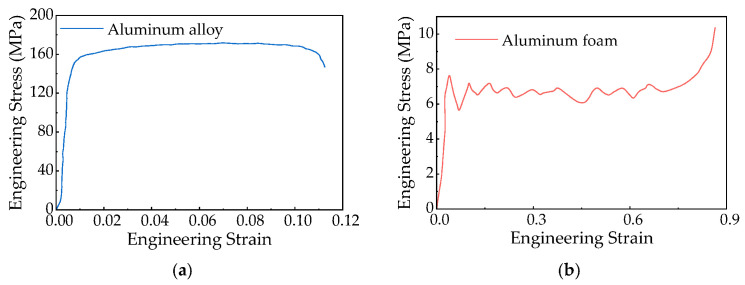
Stress–strain curves of the materials, (**a**) the aluminum alloy, (**b**) the aluminum foam.

For materials, the thin tube employs AA6063-T5 aluminum alloy with excellent fatigue resistance, while the porous filler utilizes aluminum foam with a density of 0.3 g/cm^3^ and stable energy absorption properties [44]. The thin tubes were modeled using a nonlinear elastoplastic material model to account for the large deformation range possible during impact. The foam aluminum material was modeled using Abaqus’ built-in crushable foam model to simulate its energy absorption characteristics under axial impact. The specimen preparation and testing process for the thin tube and foam aluminum are illustrated (Figure A1), with measured material parameters summarized (Figure A2 and Table 1). Although these parameters originate from small-strain experiments, the applicability of the material model under large deformation conditions is ensured by incorporating strain hardening and buckling corrections, combined with nonlinear geometric modeling (Large Deformation). Simultaneously, preliminary experiments validated the accuracy of the simulation methodology.

### 3.2. Simulation of Bionic Centipede Structures

**Figure 6:**
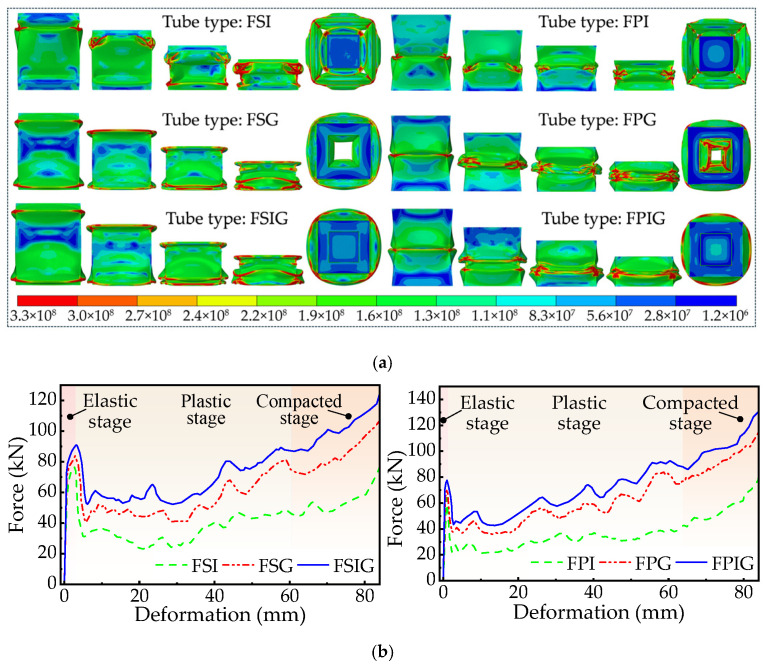
Simulation results for six structures. (**a**) Deformation process, (**b**) Force-displacement curve.

The model sizes of the six centipede-inspired structures during axial impact correspond with Section 2.2. The combination of their buckling deformation patterns and force-displacement curves (Figure 6a,b) facilitates the whole deformation process being categorized into three stages. The first phase is the elastic stage: upon initial impact, the tubular material experiences elastic deformation, causing the load to swiftly ascend to an initial peak. Subsequently, the plastic stage starts as follows: following the attainment of peak load, a marked decline transpires, succeeded by frequently occurring variations as the structure endures substantial plastic deformation. At last, the compacted stage is attained: as folds perpetually amass, the tubular body approaches densification, the response force progressively increases, and the structure demonstrates stable load-bearing properties. The above deformation stage division and mechanical response characteristics are consistent with the existing relevant research results [49,50].

**Figure 7:**
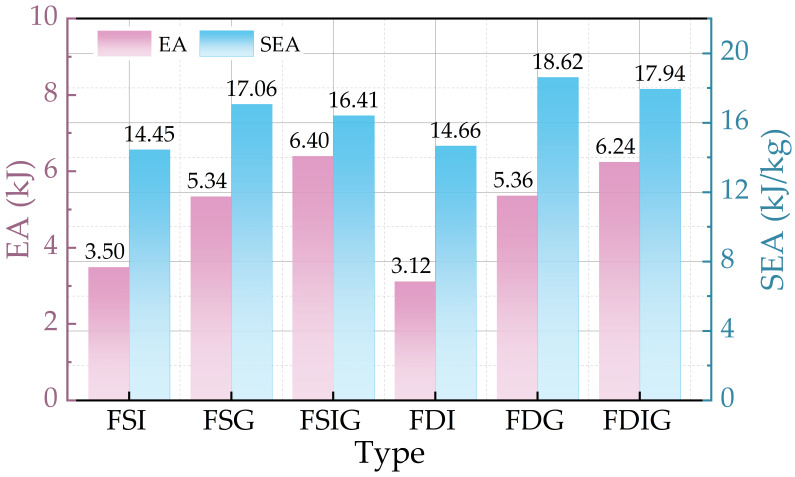
EA and SEA of six structures.

**Figure 8:**
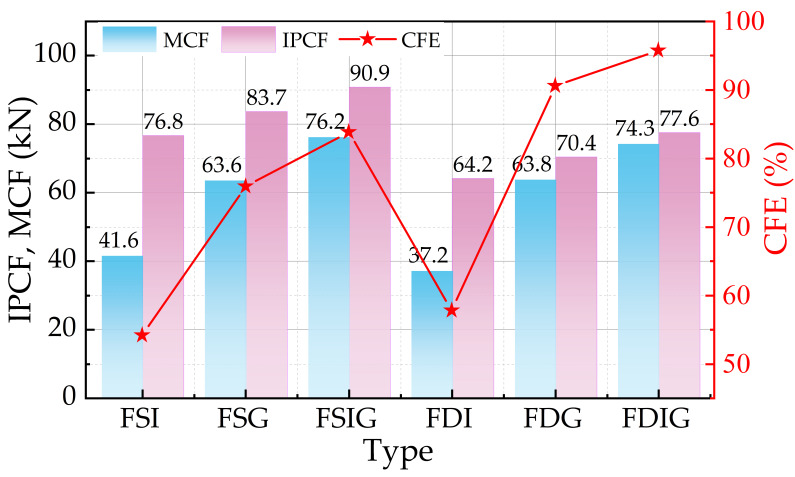
IPCF, MCF and CFE of six structures.

Six biomimetic structures were statistically analyzed for energy absorption performance based on crashworthiness assessment criteria (see Table 2), and bar charts displaying indicators like EA, SEA, IPCF, MCF, and CFE were produced (see Figure 7 and Figure 8). The results show that, (1) the EA, IPCF, and MCF values of the bidirectional pyramidal configuration were a little lower than those of the square tube structure when the three sets of comparable structures—FSI/FPI, FSG/FPG, and FSIG/FPIG—were compared. This disparity was primarily caused by the square tube’s larger volume of foam aluminum filling, which increased its total mass and, in turn, affected its total energy absorption value. However, the bidirectional pyramidal structures’ SEA and CFE values were much higher, indicating higher energy absorption efficiency per mass and more noticeable lightweight energy absorption advantages. (2) Gap-filled structures performed much better than internally filled structures when it came to foam filling sites. In square tube structures, FSG’s IPCF is 8.32% higher than FSI’s, and SEA is about 15.33% higher. FPG’s IPCF is 8.8% higher than FPI’s in bidirectional pyramidal structures, and its SEA increases by 21.28%, suggesting improved energy absorption efficiency. Gap-filled types (FSG, FPG) show slightly lower EA than both-filled types (FSIG, FPIG) when the impacts of filling techniques on performance are further compared; nevertheless, their SEA values are higher, rising by around 4.0% and 3.8%, respectively. This implies that gap-filling methods may reduce peak crushing force while enhancing certain energy absorption capacities. The remarkable lightweight and energy-absorbing characteristics of the FPG-type biomimetic centipede structure make it a strong candidate for further research in energy-absorbing structures.

### 3.3. Verification of Quasi-Static Compression in FPG Structures

Based on the simulation analysis results in Section 3.2, among the six designed centipede-inspired structural configurations, the FPG-type structure demonstrated outstanding deformation characteristics and energy-absorption performance under simulated compression conditions. This was attributed to its unique bidirectional pyramidal geometry and relatively low foam aluminum filling ratio, enabling synergistic coupling between the inner and outer tube walls. Therefore, the FPG structure is more suitable as a representative configuration for further experimental validation studies. Consistent with the approach in previous related studies [46,51,52], this research selected the FPG structure, which best represents energy-absorbing behavior, for axial quasi-static compression experiments to validate the rationality of the numerical model.

**Figure 9:**
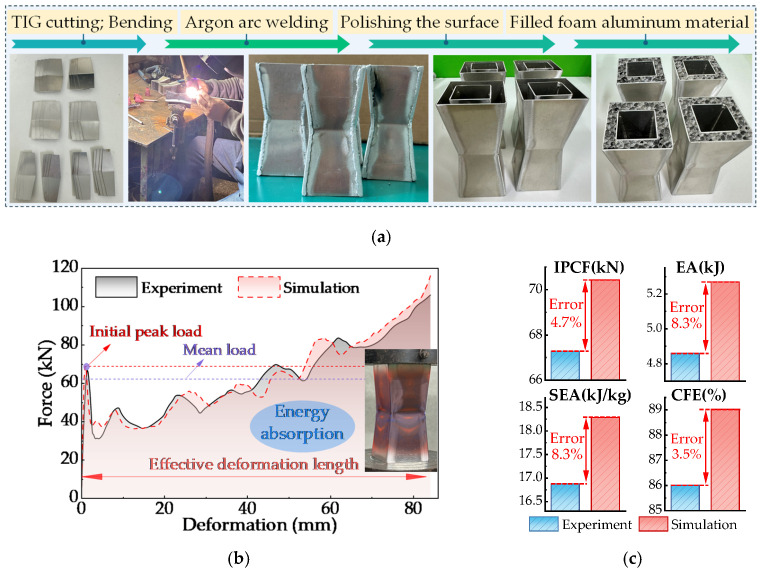
Quasi-static compression verification of FPG structure. (**a**) Fabrication of FPG specimen; (**b**) Force-displacement curve of FPG tube; (**c**) Impact resistance of FPG tube.

The experimental specimen dimensions maintain a 1:1 consistency with the finite element model. The specimen fabrication process included TIG laser cutting, bending forming, TIG welding assembly, surface polishing, and foam aluminum filling, as detailed in Figure 9a. The internal foam core utilizes a custom closed-cell foam aluminum substrate supplied by Shanghai Cici Foam Co., Ltd(Shanghai, China). It is laser-cut to achieve the required shape and dimensions, with a material density of 0.3 g/cm^3^. To ensure experimental stability and reproducibility, four independent tests were conducted on the FPG structural specimens. The results were statistically analyzed using their average values (Figure 9b,c).

The quasi-static compression test was conducted on an INSTRON 3382 electro-hydraulic servo universal testing machine (maximum load 100 kN) with a strain rate set at 10^−2^ s^−1^. Load and displacement data were collected synchronously, while an industrial camera recorded the evolution of buckling and wrinkling in the specimen during compression (Figure 9b). The entire experimental procedure was conducted in accordance with ISO 13314:2011 [53].

Experimental results demonstrate good agreement between numerical simulations and experimental data regarding initial mechanical response and peak crushing force. During the early compression phase, the load rapidly reaches its peak before exhibiting a significant decline. Subsequently, as the structure progressively undergoes wrinkling deformation, the load curve displays approximately five peaks corresponding to successive stages of structural collapse, ultimately entering a relatively stable energy absorption phase. Further comparative analysis of impact resistance metrics (Figure 9c) reveals relative errors of 4.7%, 8.3%, 8.3%, and 3.5% for the IPCF, EA, SEA, and CFE values, respectively. These results demonstrate that the established finite element model can accurately reflect the deformation patterns and energy absorption performance of the centipede-inspired structure under quasi-static compression conditions.

## 4. Parametric Analysis of FPG Under Axial Compression

This section systematically analyzes the influence of geometric parameters on the deformation behavior and impact resistance of the FPG-type centipede-inspired energy-absorbing structure. The core geometric parameters of the FPG structure include tilt angle *θ*, wall thickness *t*, foam aluminum density *ρ*, and inner tube width *d*. The wall thickness *t* was selected within the range of 0.5–2.0 mm, referencing research literature [54,55,56] on thin-walled aluminum alloy energy-absorbing structures to balance structural stiffness and toughness requirements. The aluminum foam density varied between 0.1 and 0.5 g/cm^3^. Existing studies [11,57] indicate that aluminum foam within this density range exhibits excellent impact energy absorption properties, effectively balancing energy dissipation and structural deformation. The inclination angle *θ* is set between 75° and 90°, referencing literature on optimizing the crashworthiness of conical tubes under impact loads [58] to ensure research validity. The range for inner tube width *d* is constrained by material thickness and structural geometry: considering the minimum foam aluminum plate thickness of 3 mm (thinner plates are brittle and compromise hardness), the maximum inner tube width is calculated as 74 mm based on outer tube dimensions and wall thickness, while the minimum is 34 mm, derived from geometric relationships and material constraints. To ensure comprehensive parameter analysis, 5–6 equidistant values were designed within each parameter range (see Table 3), providing a complete design space for subsequent numerical simulations and experimental validation.

### 4.1. Effect of Inclination Angle on Crashworthiness

**Figure 10:**
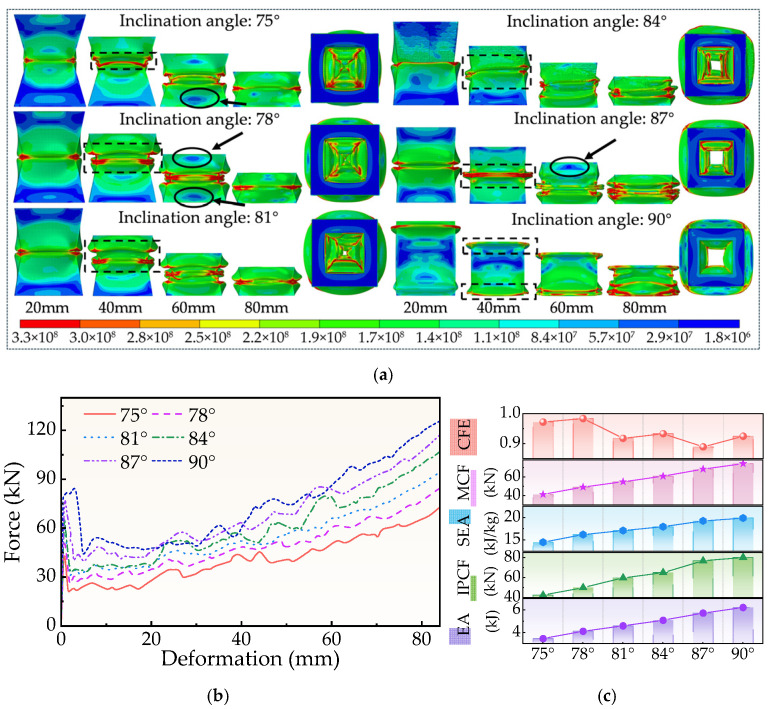
Simulation results for different θ. (**a**) Deformation process, (**b**) Force-displacement curve, (**c**) Crashworthiness index.

The development of deformation in FPG structures exposed to axial compression between 20 and 80 mm at different inclination degrees is shown in Figure 10a. The location of the plastic hinge’s formation gradually moves toward both ends of the structure as *θ* increases. Structures with slopes of 84° and 87° show significant downward displacement and buckling zone expansion at 60 mm. The impact-bearing zone expands, and when *θ* exceeds 87°, the structure’s ability to concentrate energy absorption diminishes because local component stress becomes too laterally dispersed. Additionally, a low-stress area can be apparent at the position of 60 mm when *θ* is less than 78° or greater than 87°. The local minimum stress is even lowered to about 1.8 × 10^7^ N, demonstrating that the structure has an irregular stress phenomenon that affects the stability of its energy absorption behavior.

The matching force-displacement curve is shown in Figure 10b. After encountering the initial peak crushing force, the structure of each inclination angle generally reaches an extremely steady energy absorption stage. The instability of the buckling mode and the discontinuity of the plastic energy dissipation route are reflected in the curve’s more frequent peaks and troughs when *θ* < 78°. Conversely, a prolonged high-load platform occurs before the first peak force when *θ* = 90°, indicating that the structure forms a broad pushed compression zone in the early stage. The energy absorption effect of energy-absorbing components is not supported by this very high and continuous load level.

Key crashworthiness index changes are outlined in Table A1 and Figure 10c. As the inclination angle *θ* increases, IPCF, EA, SEA, and MCF all exhibit an upward trend. Specifically, at *θ* = 75°, their values are 42.44 kN, 3.47 kJ, 14.50 kJ/kg, and 41.26 kN; at *θ* = 90°, their values are 80.12 kN, 6.22 kJ, 19.88 kJ/kg, and 74.07 kN. Throughout this process, IPCF increased by 88.76%, EA increased by 79.55%, SEA increased by 37.10%, and MCF increased by 79.55%. The primary reasons are: (1) The increased tilt angle increases the extent of material available for plastic energy dissipation through increasing the aluminum foam filling area; (2) The external thin-walled shell’s increased tilt angle boosts axial load-bearing capacity, which improves the peak fracture force in proportionally. However, when the inclination angle is too large, the overall deformation mode of the structure will be transformed into rigid resistance, resulting in the inhibition of the progressive collapse characteristics that the energy-absorbing structure should have, thereby reducing its energy absorption effect.

In summary, the inclination angle has a dual effect on the energy absorption performance of the FPG structure. Too small inclination angle will lead to buckling instability and discontinuous stress concentration, while too large inclination angle will make the stress mode of the structure tend to be rigid, thus reducing the flexible collapse capacity required in the energy absorption process. Therefore, the optimal energy absorption performance of the FPG structure usually appears in a moderate inclination angle range, and a reasonable balance needs to be found between the stable buckling mode and the effective energy absorption capacity.

### 4.2. Effect of Wall Thickness on Crashworthiness

**Figure 11:**
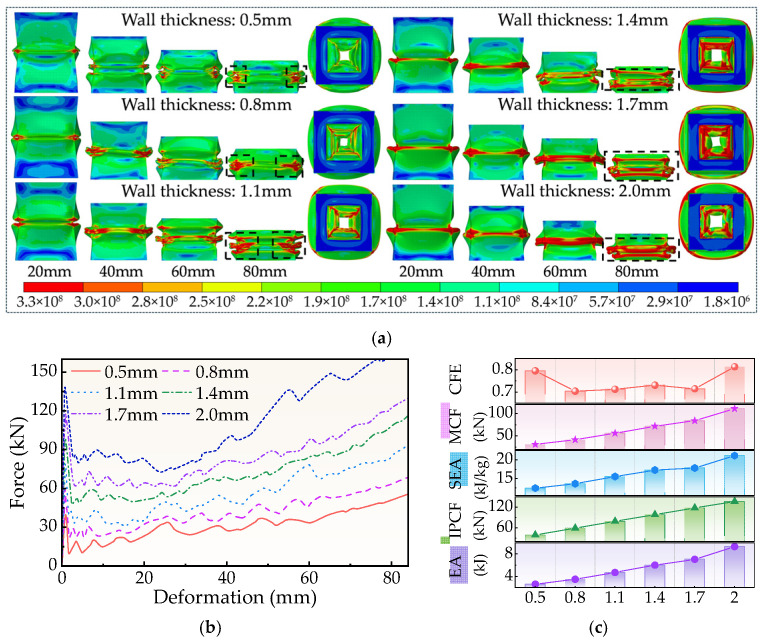
Simulation results for different t. (**a**) Deformation process, (**b**) Force-displacement curve, (**c**) Crashworthiness index.

One important factor influencing the FPG energy absorption unit’s general stability, initial peak force, and energy absorption mode is the wall thickness t. Figure 11a shows how the local buckling sensitivity of thin-walled tubes reduces as wall thickness grows, and the structure progressively switches from “local wrinkle-dominated” to “overall collapse-dominated.” The structure has outstanding deformation controllability and is readily capable of creating buckling waves locally under thin-walled conditions (*t* = 0.5 mm), which leads energy to dissipate rapidly in a small area. Increasing the wall thickness to 2.0 mm greatly improves the tube wall’s stiffness, which causes stress to be transferred over a larger area during compression. As a result, the high-stress band almost entirely covers the loading end, preventing the formation of controllable wrinkles. The lack of stability leap that commonly follows such “overall rigid crushing” shrinks the energy absorption process’s smoothness.

The initial peak force grows greatly with wall thickness (Figure 11b), reaching from 39.69 kN for 0.5 mm to 136.85 kN for 2.0 mm), the increase rate is 245%. In addition to increasing impact acceleration during the structure’s startup phase, the accompanying peak force surge could lead to an in-load transfer that is unfavorable to the main structure, even though thicker walls can increase the plastic stage’s bearing capacity and cause the total energy absorption EA to continue increasing. Further, improving the energy absorption capacity per unit mass is constrained by the mass increase brought on by thicker walls.

Table A2 and Figure 11c further demonstrate that while an increase in wall thickness raises EA from 2.65 kJ to 9.35 kJ, an increase in mass from 0.212 kg to 0.441 kg limits the rise in SEA value. It means that while the wall thickness is too large, the rate of energy absorption decreases due to the simultaneous increase in mass and peak force. In contrast, the FPG construction still has room to maximize the energy absorption efficiency in the thin-walled interval (≤1.0–1.2 mm).

In short, wall thickness is an important factor that impacts the buckling mode, energy distribution mode, and energy absorption efficiency as well as being the primary parameter influencing the structural strength. Under the presumption of ensuring structural strength and stability, the correct choice of wall thickness may achieve the optimum equilibrium between mass, peak force, and energy absorption efficiency.

### 4.3. Effect of Foam Density on Crashworthiness

**Figure 12:**
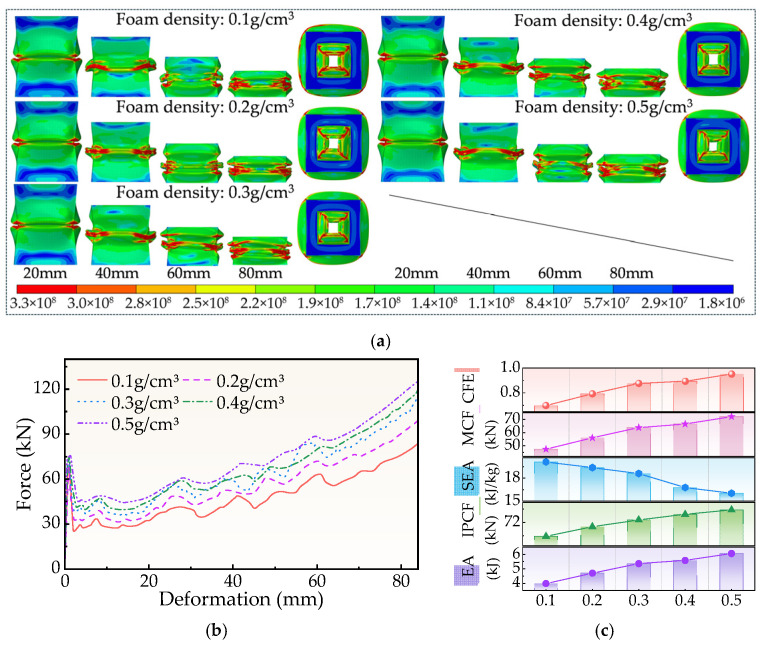
Simulation results for different *ρ*. (**a**) Deformation process, (**b**) Force-displacement curve, (**c**) Crashworthiness index.

The impact of aluminum foam density *ρ* on the FPG structure’s deformation mode is portrayed in Figure 12a. The foam material provides stronger support for the thin-walled outer tube during the compression process, the plastic hinge formation is more stable, the buckling position becomes simpler to control, and the overall stress field is more uniform as *ρ* grows since it increases the foam material’s yield platform stress. The high-density foam enhances the stability of deformation and eliminates early local wrinkles by strengthening the synergy between the outer tube and the interior filler material.

The force-displacement curve in Figure 12b shows that the structure exhibits a similar stage buckling mode at different densities, but the high-density foam can increase the platform load and increase the EA value. This is mainly due to the enhanced interfacial force transfer ability between the foam and the tube wall after the density increase.

However, as shown in Figure 12c and Table A3, with increasing density, the IPCF value increased from 67.86 kN to 75.78 kN, representing an 11.67% change. Conversely, the SEA value decreased from 20.14 kJ/kg to 16.03 kJ/kg, reflecting a 20.41% reduction. The main reason is that the structural quality is much improved by the density increase, which dilutes the energy absorption efficiency per unit mass. Also, it might shorten the time for effective energy absorption and lead the foam to enter the densification stage earlier.

In conclusion, a moderate increase in foam density enhances bearing capacity and overall energy absorption; yet, an excessively high density could result in negative effects like higher weight and decreased SEA. Therefore, aluminum foam’s density *ρ* is more suited as a controlled parameter to balance mass and energy absorption performance than as the main optimization variable of FPG.

### 4.4. Effect of Inner Tube Width on Crashworthiness

**Figure 13:**
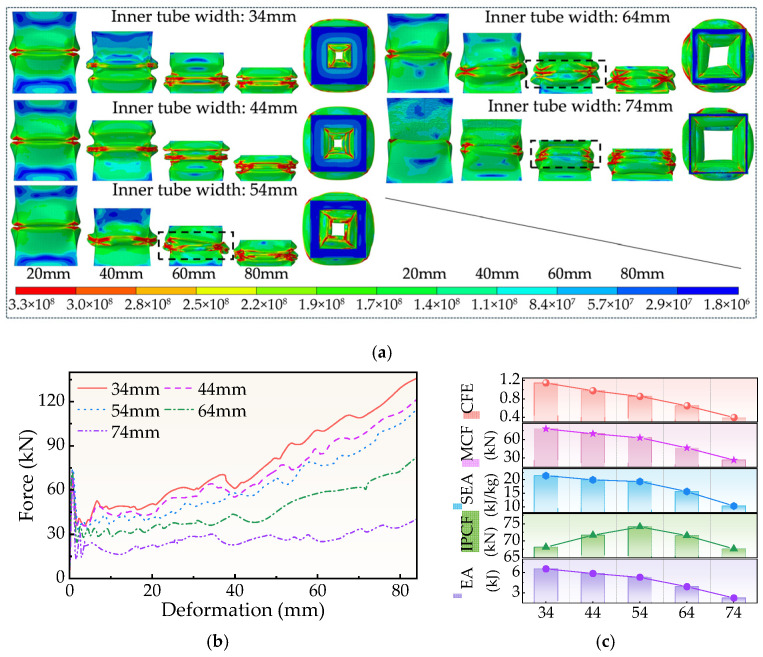
Simulation results for different d. (**a**) Deformation process, (**b**) Force-displacement curve, (**c**) Crashworthiness index.

The FPG structure’s deformation mode under various inner tube widths (*d*) appears in Figure 13a. The structure exhibits obvious asymmetric collapsing at approximately 60 mm displacement when *d* grows to 54 mm and 64 mm. Moreover, the folding transfers to one side, leading to overall instability. The efficiency of energy absorption will be reduced by this inequality distortion. On the other hand, the structure always retains satisfactory symmetry when *d* falls between 34 and 44 mm, and the synergistic deformation of aluminum and thin-walled foam is stable, facilitating the emergence of a controllable wrinkle mode.

The force-displacement curve in Figure 13b shows that as *d* increases, the load rise rate slows down and the energy absorption capacity decreases. The shorter *d* shows a steeper force rise curve, indicating that its energy absorption is more sufficient.

Figure 13c and Table A4 further validate this trend: IPCF first increases and then decreases with increasing d. At *d* = 34 mm, IPCF is 68.15 kN; at *d* = 54 mm, IPCF increases to 74.17 kN; and at *d* = 74 mm, IPCF decreases to 67.67 kN. In contrast, SEA, MCF, and CFE all exhibit a continuous decreasing trend with increasing *d*. This indicates that excessively large *d* values weaken stable energy dissipation capacity and induce unstable collapse.

Therefore, *d* is a key parameter affecting the structural performance of FPG. The appropriate length of the inner tube (about 34–44 mm) can ensure high energy absorption efficiency while maintaining symmetrical buckling, while too large *d* is not conducive to structural optimization.

## 5. Multi-Objective Optimization of FPG Structures

Section 4, the influence of geometric parameters (*θ*, *ρ*, *d*, *t*) on the energy absorption performance of FPG structure is systematically analyzed. The results show that *ρ* has little effect on the performance, while *θ*, *d* and *t* have more significant effects on energy absorption, buckling morphology and quality control. Based on this, the Section 5 selects *θ*, *d* and *t* as the main optimization variables to carry out multi-objective optimization design with the goal of improving energy absorption performance and reducing peak crushing force.

### 5.1. Optimization Methods

This study selected the highly representative impact resistance metrics SEA and IPCF to further enhance the energy absorption capacity of the energy-absorbing structure [16,59,60]. Simultaneously, to reduce computational costs, other standards such as EA, CFE, and MCF were excluded. Consequently, the optimization design objective was transformed into maximizing SEA and minimizing IPCF, with the mathematical expression as follows:

(1) $$\left\{\begin{array}{c} \begin{array}{c} F(x)=max\left\{SEA\left.\left(\theta ,d,t\right)\right\}\right.\\ F(x)=min\left\{IPCF\right.\left.\left(\theta ,d,t\right)\right\}\\ \theta \in \left[75^{{}^{\circ}}{,90}^{{}^{\circ}}\right],d\in \left[34mm,74mm\right],t\in \left[0.5mm,2.0mm\right] \end{array} \end{array}\right.$$

where *F(x)* is the objective function, and *θ*, *d* and *t* are design variables. The key to optimization is to reasonably select structural parameters under geometric constraints, achieve a balance between energy absorption performance and lightweight, and improve structural safety and material utilization.

In recent years, optimal Latin hypercube sampling (OLHS) has been widely used because of its uniform spatial distribution and low correlation of variables. KRG model shows excellent prediction and generalization ability in high-dimensional nonlinear systems. The third-generation non-dominated sorting genetic algorithm (NSGA-III) has become an important means of multi-objective optimization with its excellent Pareto solution distribution and dimensional adaptability.

**Figure 14:**
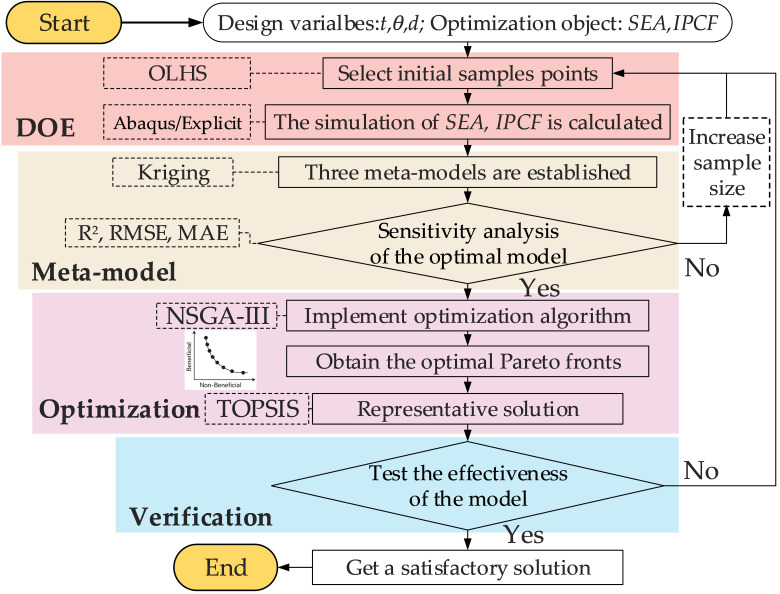
Flowchart of the optimization design.

Based on this, this paper constructs a set of multi-objective optimization process suitable for collision energy absorption structure: first, 40 groups of samples are extracted in the parameter space by using OLHS; then the finite element analysis is carried out by Abaqus/Explicit to obtain the SEA and IPCF indexes. Then, the KRG meta-model is established, and the sensitivity analysis is carried out. Finally, multi-objective optimization was achieved using NSGA-III to obtain the Pareto frontier, and the optimal solution closest to the ideal point was selected via the TOPSIS method (Figure 14).

### 5.2. Optimization Results

**Figure 15:**
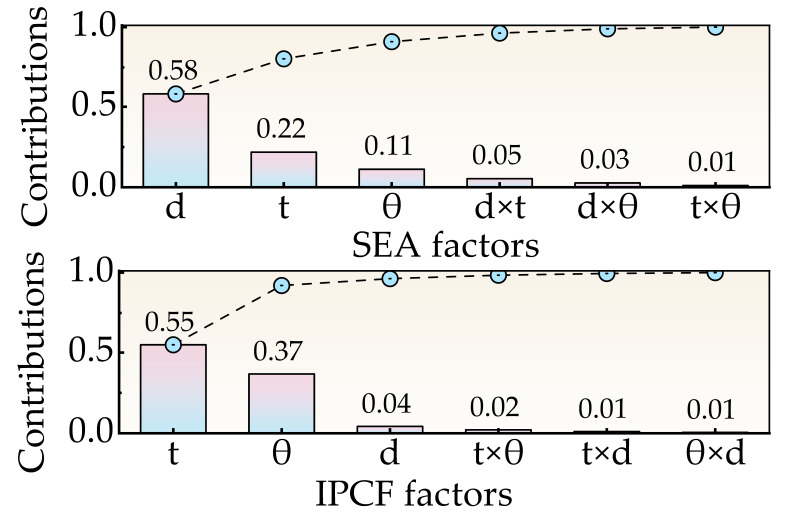
The influence bar chart of SEA and IPCF values are output.

The OLHS method is implemented using the ‘pyDOE’ library in Python 3.13, and the generated 40 sets of uniformly distributed sample points are shown in Table A5. Subsequently, each sample was simulated in Abaqus/Explicit to extract SEA and IPCF results (see Table A5). Based on the obtained data, the KRG model is used to train the objective function (SEA and IPCF), and the model accuracy indicators R^2^, RMSE and MAE (Table 4) are calculated. The results show that the model prediction accuracy is good and the error is within a reasonable range. Figure 15 shows the sensitivity analysis results of SEA and IPCF. The sum of the main effects and interactions of variables is close to 1, which further verifies the reliability of the model.

**Figure 16:**
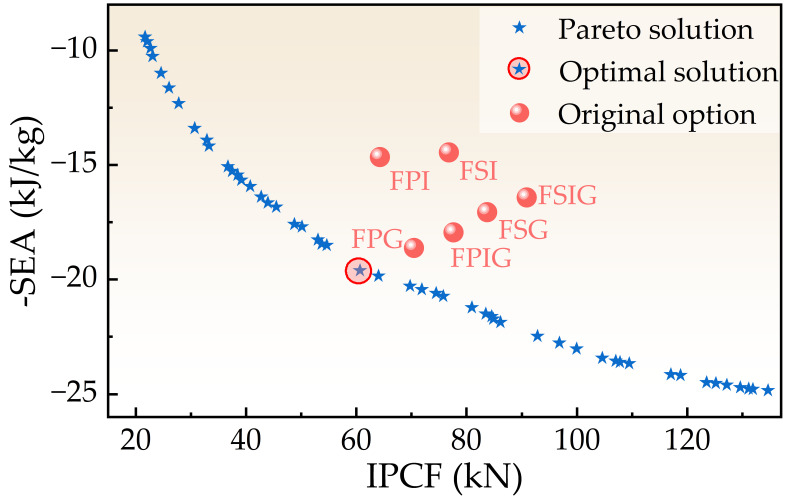
Pareto frontier of optimized structures.

Subsequently, the NSGA-III algorithm was used to carry out multi-objective optimization. The parameters were set as follows: population size 300, maximum number of iterations 300, crossover probability 0.9, and mutation probability 0.33. Based on the joint operation of MATLAB R2024b Optimization Toolbox and Kriging surrogate model, 40 groups of evenly distributed Pareto optimal solutions (Table A6) are obtained, and the optimal solution was identified using the TOPSIS method. As shown in Figure 16, the comparison between the optimized solution set and the initial structure shows that when *θ* = 86.25°, *d* = 35.24 mm, *t* = 0.665 mm, the SEA value of the FPG structure is increased to 19.6 kJ/kg, and the IPCF value is reduced to 61.65 kN. Compared with the original structure, the overall performance is improved by 5.28% in SEA and reduced by 13.9% in IPCF, which effectively takes into account the energy absorption capacity and structural safety, reflecting the significant advantages of optimization design.

### 5.3. Comparison and Validation of Structural Improvements Before and After

**Figure 17:**
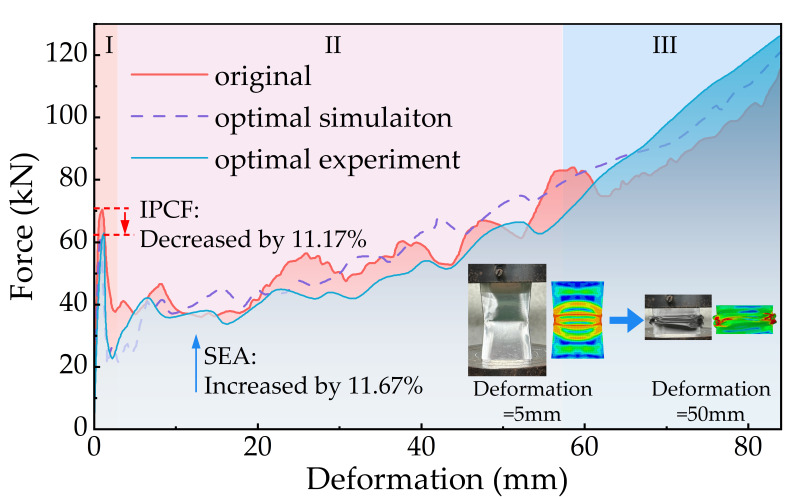
Force-displacement curves before and after optimization.

For the optimal FPG structure obtained in Section 5.2, a corresponding finite element simulation model was further established. A physical specimen was fabricated to conduct quasi-static compression tests, aiming to validate the optimization results while systematically analyzing its deformation evolution and failure mechanism. As shown in Figure 17, the mechanical response of the structure during the collapse process can be divided into three typical stages.

In Stage I, both the original structure and the optimized structure (simulation results and experimental results) rapidly undergo global plastic buckling and form an initial failure mode. Compared to the original FPG structure with an IPCF of 70.44 kN, the optimized structure exhibits an IPCF of approximately 60 kN, representing a reduction of about 11.17%. This result indicates that the optimized structure effectively mitigates load surges during the initial loading phase, producing a smoother impact response. This improvement enhances the safety performance of the energy-absorbing structure during the early stages of impact.

In Stage II, the load–displacement curves of all three structures exhibited an overall fluctuating upward trend, yet their stability showed significant differences. The original FPG structure experienced multiple pronounced load peaks and troughs during this stage, indicating pronounced instability in its buckling process. In contrast, the load increase process for the optimized simulated and experimental structures was more continuous, with stable and uniform energy absorption, demonstrating superior deformation controllability and energy absorption stability.

Upon entering Stage III, the structure gradually transitions into a densification phase. At this point, the optimized FPG structure exhibits significantly enhanced energy absorption capacity. During sustained compression, the foam aluminum material undergoes continuous fragmentation and forms an effective synergistic deformation mechanism with the outer thin-walled components, thereby substantially improving the overall energy absorption efficiency of the structure.

Further comparative analysis results are shown in Table 5. While the IPCF value decreased by 11.17%, the SEA value of the optimized structure increased by 11.67%. Additionally, the prediction errors between the optimized model and experimental test values were 1.50% and 3.78%, respectively, both within acceptable ranges. This fully validates the reliability of the proposed FPG structural optimization design method in terms of numerical prediction accuracy and engineering applicability.

## 6. Conclusions

In this study, a bidirectional pyramidal energy-absorbing structure that mimics the shape of the centipede was designed based on the foam-filled structure and bionic design theory. The crashworthiness of the structure was systematically evaluated by experiments, theoretical analysis and numerical simulation. The main conclusions are as follows:

(1) The experimental results are in good agreement with the finite element simulation results. The relative errors of IPCF, EA, SEA and CFE are 4.7%, 8.3%, 8.3% and 3.5%, respectively, and the overall error is less than 7%, which indicates that the established simulation model has high reliability and is suitable for the follow-up study of this kind of bionic structure.

(2) Among the six structures, the aluminum foam-filled bi-directional pyramid gap tube (FPG) exhibits the best stability, stiffness characteristics and energy absorption efficiency. Its single factor analysis shows that the structure has better comprehensive performance and optimization potential when *θ* is in the range of 78–87°, *t* is less than 0.1 mm and *d* is in the range of 34–44 mm. However, *ρ* should not be used as a design variable for subsequent optimization.

(3) The optimization scheme realizes the SEA value is increased by 11.67%, and the IPCF is reduced by 11.17%. The prediction error of each performance index is less than 5%, which indicates that this optimization method can be compatible with structural lightweight and crashworthiness optimization.

In summary, FPG structures demonstrate promising applications in the field of collision safety for transport equipment, including vehicles, aviation, and marine engineering. Although this study achieved positive results in optimizing energy-absorbing structures, certain limitations remain. For instance, verification has not yet been performed on all structures, the current optimization objectives fail to encompass all factors potentially affecting structural crashworthiness, and the employed optimization strategy is relatively limited, insufficiently accounting for the impact of diverse operational conditions. Future research may explore integrating polygonal cross-section elements with lattice or honeycomb structures to conduct multiscale modeling analysis. This approach aims to enhance structural crashworthiness and engineering applicability.

## Figures and Tables

**Table 1:** Materials properties of components in structure.

Materials	Density (g/cm^3^)	Tensile Modulus (MPa)		Poisson’s Ratio		Yield Stress (MPa)					
						Tensile		Compression		Shear	
		Avg	S_R_	Avg	S_R_	Avg	S_R_	Avg	S_R_	Avg	S_R_
Foam	0.3	130	21	0.01	0.005	3.82	0.8	3.87	0.25	2.4	0.3
6063T5	2.7	70,000	3000	0.31	0.012	138.7	11	-	-	-	-

**Table 2:** Energy absorption performance of different types of tubes.

Type	M (kg)	EA (kJ)	IPCF (kN)	SEA (kJ/kg)	MCF (kN)	CFE (%)
FSI	0.242	3.50	76.76	14.45	41.62	54.22
FSG	0.313	5.34	83.73	17.06	63.58	75.94
FSIG	0.39	6.40	90.90	16.41	76.20	83.83
FPI	0.213	3.12	64.24	14.66	37.16	57.85
FPG	0.288	5.36	70.44	18.62	63.83	90.62
FPIG	0.348	6.24	77.62	17.94	74.32	95.76

**Table 3:** Parametric analysis variable design domain.

Parameters	Initial Value	Design Domain	Intervals	Unit
*θ*	85	[75, 90]	3	(°)
*ρ*	0.3	[0.1, 0.5]	0.1	g/cm^3^
*d*	48	[34, 74]	10	mm
*t*	1	[0.5, 2]	0.3	mm

**Table 4:** Accuracy and error parameters of the objective function.

	SEA	IPCF
R^2^	0.981	0.9938
RMSE	0.4929	2.2239
MAE	0.3912	1.8546
RMSE/mean (y)	0.02934754	0.028673436
MAE/mean (y)	0.02329227	0.023911936

**Table 5:** Results of optimization compared to the original design.

Style	*θ* (°)	*d* (mm)	*t* (mm)	SEA (kJ/kg)	IPCF (kN)
Basic FPG	85	48	0.5	18.617	70.44
Optimal model	86.25	35.24	0.665	19.6	60.65
Optimal simulation	86.25	35.24	0.665	20.79	62.57
Optimal experiment	86.25	35.24	0.665	19.89	62.94
Error of model and experiment				1.50%	3.78%
Comparison before and after optimization				↑ 11.67%	↓ 11.17%

"↑" represents an increase, "↓" represents a decrease.

## Data Availability

The original contributions presented in this study are included in the article/Supplementary Material. Further inquiries can be directed to the corresponding authors.

## Supplementary Materials

The following supporting information can be downloaded at: https://www.mdpi.com/article/10.3390/biomimetics11010046/s1, Equations (S1)-(S4).

## Appendix A

This section primarily supplements the experimental details and data presented in the main text.

**Table A1:** Energy absorption performance of tubes at different inclination angles.

Type	M (kg)	EA (kJ)	IPCF (kN)	SEA (kJ/kg)	MCF (kN)	CFE (%)
75°	0.239	3.47	42.44	14.50	41.26	97.20
78°	0.254	4.12	49.84	16.21	49.03	98.36
81°	0.269	4.6	59.65	17.10	54.75	91.79
84°	0.284	5.09	64.99	17.94	60.65	93.32
87°	0.298	5.73	76.74	19.24	68.25	88.94
90°	0.313	6.22	80.12	19.88	74.07	92.46

**Table A2:** Energy absorption performance of tubes with different wall thicknesses.

Thickness (mm)	M (kg)	EA (kJ)	IPCF (kN)	SEA (kJ/kg)	MCF (kN)	CFE (%)
0.5	0.212	2.65	39.6918	12.51	31.57851	0.795593
0.8	0.258	3.53	59.6574	13.69	42.05327	0.704913
1.1	0.304	4.75	79.213	15,615.44	56.51301	0.713431
1.4	0.349	6.06	98.5608	17,351.06	72.08953	0.731422
1.7	0.395	7.08	117.768	17,911.46	84.22649	0.71519
2	0.441	9.35	136.848	21,206.48	111.334	0.81356

**Table A3:** Energy absorption performance of tubes with different foam densities.

Density (g/cm^3^)	M (kg)	EA (kJ)	IPCF (kN)	SEA (kJ/kg)	MCF (kN)	CFE (%)
0.1	0.198	3.99	67.86	20.14	47.47	69.96
0.2	0.243	4.71	70.70	19.39	56.10	79.35
0.3	0.288	5.36	72.77	18.62	63.83	87.72
0.4	0.333	5.59	74.43	16.79	66.54	89.40
0.5	0.378	6.06	75.77	16.03	72.13	95.19

**Table A4:** Energy absorption performance of tubes with different d values.

*d* (mm)	M (kg)	EA (kJ)	IPCF (kN)	SEA (kJ/kg)	MCF (kN)	CFE (%)
34	0.306	6.56	68.15	21.43	78.05	114.53
44	0.295	5.88	71.68	19.92	69.94	97.58
54	0.276	5.32	74.17	19.27	63.31	85.36
64	0.251	3.93	71.58	15.64	46.73	65.29
74	0.218	2.26	67.67	10.38	26.94	39.82

**Table A5:** Sampling values and model output results.

No.	*θ* (°)	*d* (mm)	*t* (mm)	SEA (kJ/kg)	IPCF (kN)	No.	*θ* (°)	*d* (mm)	*t* (mm)	SEA (kJ/kg)	IPCF (kN)
1	79.75	38.38	0.676	16.54	40.36	21	78.53	72.81	1.104	8.40	50.57
2	80.75	53.31	0.697	12.48	41.56	22	78.77	60.99	0.590	10.14	31.06
3	85.80	67.08	1.164	10.74	89.76	23	89.75	49.89	1.777	15.90	139.84
4	75.63	69.20	1.990	13.50	83.70	24	89.54	37.25	1.268	17.34	93.80
5	83.77	35.68	1.525	18.36	94.54	25	82.96	34.53	0.896	17.59	57.17
6	85.27	36.29	1.304	17.49	86.77	26	88.88	51.22	1.599	15.35	123.64
7	83.50	52.04	0.995	14.28	67.34	27	82.05	70.75	1.693	11.90	108.07
8	81.70	50.91	1.662	16.39	100.05	28	87.84	43.05	1.183	16.85	87.14
9	87.14	48.54	1.822	17.52	131.23	29	84.31	46.81	0.737	16.07	52.92
10	86.64	54.70	0.515	13.69	42.70	30	82.55	47.38	1.352	15.51	84.87
11	85.05	42.43	1.486	16.97	100.95	31	87.71	40.95	1.957	19.47	136.30
12	77.52	45.46	0.767	14.23	40.63	32	77.86	59.75	1.224	12.55	59.24
13	86.01	62.42	1.738	14.72	131.51	33	82.32	71.28	1.908	12.34	126.23
14	88.20	73.12	0.615	6.45	51.19	34	80.59	57.03	1.386	14.86	79.97
15	76.59	68.89	1.706	12.59	73.31	35	88.86	58.88	0.934	11.46	75.41
16	81.11	39.50	0.965	16.42	56.99	36	80.05	66.36	1.067	10.38	56.86
17	86.31	44.17	0.545	17.36	44.53	37	78.25	56.08	1.854	16.20	92.48
18	79.42	55.95	1.053	12.98	56.93	38	84.73	64.12	1.464	13.10	107.52
19	75.34	61.40	0.856	10.23	36.29	39	77.21	65.58	0.832	9.47	36.76
20	76.05	41.76	1.566	16.79	65.85	40	76.17	63.17	1.424	12.40	60.36

**Table A6:** 50 generations of pareto cutting-edge solutions.

No.	*θ* (°)	*d* (mm)	*t* (mm)	SEA (kJ/kg)	IPCF (kN)	No.	*θ* (°)	*d* (mm)	*t* (mm)	SEA (kJ/kg)	IPCF (kN)
1	86.14	34.32	0.767	19.85	64.01	26	79.43	34.29	1.912	23.02	99.95
2	76.45	68.47	0.500	9.41	21.73	27	78.18	34.50	1.683	21.49	83.47
3	78.44	55.58	0.514	13.91	32.93	28	79.16	48.54	0.533	15.93	40.74
4	78.08	49.98	0.514	15.28	37.48	29	77.95	48.24	0.524	15.66	39.18
5	78.55	34.37	1.591	21.23	80.94	30	85.02	34.36	1.997	24.71	129.62
6	77.08	49.77	0.529	15.08	36.75	31	78.37	45.48	0.541	16.39	42.72
7	86.09	34.30	0.917	20.29	69.74	32	84.31	34.40	1.012	20.43	71.92
8	76.48	65.90	0.503	10.26	23.09	33	77.23	34.41	1.812	21.72	84.81
9	85.46	34.29	1.998	24.77	131.12	34	77.23	34.38	1.843	21.86	86.16
10	86.70	34.15	2.000	24.84	134.66	35	77.57	48.93	0.546	15.44	38.50
11	78.13	34.50	1.712	21.61	84.54	36	79.01	34.36	1.890	22.76	96.81
12	76.71	59.56	0.519	12.32	27.80	37	80.96	36.58	0.555	18.44	53.71
13	82.29	34.32	1.973	24.15	117.02	38	83.58	34.36	1.127	20.73	75.75
14	80.41	34.33	1.972	23.62	107.77	39	76.69	61.74	0.512	11.64	26.06
15	76.64	67.08	0.505	9.91	22.73	40	77.32	56.34	0.506	13.39	30.68
16	79.98	34.67	0.589	18.51	54.62	41	83.60	34.21	1.988	24.50	123.53
17	77.49	53.75	0.516	14.17	33.33	42	78.07	34.37	1.903	22.47	92.81
18	84.60	34.33	1.980	24.60	127.20	43	78.39	37.67	0.564	17.71	50.13
19	79.49	45.48	0.530	16.66	43.98	44	85.72	34.27	1.997	24.78	131.87
20	84.10	34.29	1.979	24.54	125.16	45	76.52	63.68	0.511	10.99	24.62
21	80.12	41.18	0.533	17.58	48.74	46	79.79	34.24	1.972	23.42	104.61
22	80.35	34.37	1.963	23.55	107.00	47	86.25	35.24	0.665	19.60	60.65
23	85.65	34.64	1.031	20.61	74.51	48	76.47	67.84	0.503	9.62	22.11
24	79.93	36.55	0.579	18.27	53.08	49	81.17	34.31	1.930	23.66	109.45
25	82.84	34.51	1.956	24.18	118.78	50	77.59	42.40	0.599	16.83	45.49
