# Supplementary material for: Crashworthiness Design of Bidirectional Pyramidal Energy-Absorbing Tubes Based on Centipede Structures
Source: Biomimetics (Basel). 2026 Jan 7;11(1):46. doi: 10.3390/biomimetics11010046 (PMC12838966; doi:10.3390/biomimetics11010046)
Supplement: Supplementary file 1 [file biomimetics-11-00046-s001.zip › Supplementary Materials.pdf]

Supplementary Materials:

The following support information is mainly the calculation formula of Section 2.3.

The initial peak crushing force (IPCF) is characterized as the first peak force observed during the structural crushing process.

EA (energy absorption) can be calculated as follows:

$$EA = \int_0^{d_{max}} F(s)ds \qquad (S1)$$

F(s) represents the crushing force as a function of displacement s throughout the crushing process, whereas dmax denotes the effective deformation distance (or effective stroke).

The mean crushing force (MCF) represents the average compressive force, which can be approximated as follows:

$$MCF = \frac{EA}{d_{max}} = \frac{\int_0^{d_{max}} F(s)ds}{d_{max}} \qquad (S2)$$

Crushing force efficiency (CFE) is defined as the quotient of MCF and IPCF. It quantifies load variations during structural failure and can be computed as:

$$CFE = \frac{MCF}{IPCF} \qquad (S3)$$

Specific energy absorption (SEA) per unit mass is calculated as follows:

$$SEA = \frac{EA}{m} \qquad (S4)$$

In the equation, m represents the mass of the energy absorber.
